# Seroprevalence of Bluetongue in sheep in Kohgiluyeh and Boyer-Ahmad province, Iran

**Published:** 2014

**Authors:** Mohammad Sabaghan, Mahdi Pourmahdi Borujeni, Masoud Reza Seifi Abad Shapouri, Aria Rasooli, Milad Norouzi, Sayeh Samimi, Siavash Mansouri

**Affiliations:** 1 *Department of Parasitology, Faculty of Medicine, Ahvaz Jondishapour University of Medical Science, Ahvaz, Iran; *; 2 *Department of Food Hygiene, Faculty of Veterinary Medicine, Shahid Chamran University, Ahvaz, Iran; *; 3 *Department of Pathobiology, Faculty of Veterinary Medicine, Shahid Chamran University, Ahvaz, Iran; *; 4 *Department of Clinical Science, Faculty of Veterinary Medicine, Shahid Chamran University, Ahvaz, Iran; *; 5 * Graduated from Faculty of Veterinary Medicine, Shahid Chamran University, Ahvaz, Iran.*

**Keywords:** Bluetongue, ELISA, Iran, Sheep

## Abstract

Bluetongue (BT) is a viral disease of ruminants transmitted by Culicoides biting midges and has the ability to spread rapidly over large distances. The disease occurs almost worldwide between latitudes approximately 35˚ S and 50˚ N. Among the numerous diseases of ruminants, BT has gained considerable importance in recent years as one of the best examples of the effects of climate change on disease spread. Sheep are major livestock species in Iran, but studies of BT have not gained the priority compared to other diseases. Thus, the objective of this study was to describe the distribution and seroprevalence of bluetongue virus (BTV) infections in sheep in Kohgiluyeh and Boyer-Ahmad province of Iran, and to identify factors associated with the exposure of these sheep to BTV infection. Sera from 262 apparently healthy sheep were collected during the year 2011. The collected sera of the animals were screened with competitive enzyme like immunosorbent assay (c-ELISA). Two hundred and three (77.48%) out of 262 sera tested were positive to BTV antibodies. Statistically significant differences were found in the seroprevalence BT, between sex and age of sheep (*p* < 0.001). No statistically significant differences were observed in BTV seroprevalence among different seasons, nor among recently aborted and normally delivered.

## Introduction

Bluetongue (BT) is an infectious disease of wild and domestic ruminants caused by bluetongue virus (BTV). The RNA virus belongs to the Orbivirus genus in the Reoviridae family. It is transmitted by biting midges of the genus Culicoides. Up to now 24 distinct serotypes of the virus have been described.^1^^,^^2^ The disease was first described in an imported Merino sheep in South Africa in the 19^th^ century. In 1902 the disease was mentioned as “a malarial catarrhal fever of sheep”, and was named as “bluetongue” in 1905.^3^ Although BTV may infect many species of ruminants, sheep are usually the most severely affected animals. Viremia in sheep and goats commence from three days post infection and may last up to 54 days.^4^ Severe disease occurs most commonly in certain breeds of sheep, but the severity of BT is highly variable.^5^

The virus causes infection and clinical disease in sheep, the primary clinical signs of BTV infection is hemorrhage and ulceration of the mucous membranes in the upper portion of the gastrointestinal tract, including the oral cavity and esophagus. Other signs such as coronitis, laminitis, facial edema, and transient infertility are also seen in sheep. Cattle rarely demonstrate clinical disease.^6^ Clinical signs may be acute and mortality can be up to 70.00% in some flocks of sheep.^7^

The historically the geographical distribution of the BT has been approximately between latitudes of 50˚ N and 35˚ S, in temperate and tropical regions of the world.^8^ This area coincides with the distribution of specific species of Culicoides midges that are biological vectors of the virus.^9^^,^^10^ Although the relationship between the virus and vector is not yet fully understood, environmental and genetic factors are important determinants of bluetongue activity within the vector and its ecosystem.^11^

**Fig. 1:**
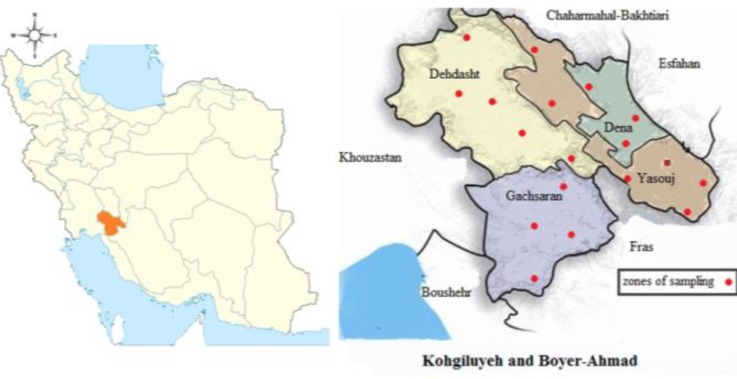
Kohgiluyeh and Boyer-Ahmad province in of Iran

While sheep are a major livestock species in Iran, studies of BT have not been given the same priority as some other diseases. Thus, the objective of this study was to describe the distribution and seroprevalence of BTV infection in sheep in Kohgiluyeh and Boyer-Ahmad province in Iran, in 2011. This province is 15504 Km^2^ and is located between latitude 30˚30´ to 31˚30´ N and longitude 51˚ to 52˚ E in the southwest of Iran (Fig. 1).

## Materials and Methods

**Sampling.** A total number of 262 serum samples were collected from apparently healthy sheep of various ages and either sexes during four seasons in the year 2011, from 20 flocks in 17 different locations in Kohgiluyeh and Boyer-Ahmad province. Age was determined by tooth replacement in sheep.^12^ The animals were divided into three age groups: juvenile (≤ 1 year old), sub-adult (1 to 3 years old) and adult (> 3 years old). The blood samples were collected from the jugular vein with sterile tubes of venoject )Zhejiang U-REAL medical technology Co., Taizhou, China) without anticoagulant and the sample shipped from zones of sampling to laboratory in dry ice and then centrifuged at 3000 rpm for 15 min. Then, serum was separated and stored at *−*20 ˚C until enzyme like immunosorbent assay (ELISA) was performed.

**Serological test. **The competitive ELISA (c-ELISA) has proved to be the best serologic test for detecting group antibodies to BTV.^13^ In this study, BTV antibody levels were measured using c-ELISA IDEXX bluetongue competition^®^ assay (IDEXX BT, Hoofddorp, The Netherlands) according to the manufacturer’s instructions. The optic density of each sample was read by an ELISA microplate reader (PowerWave XS2; BioTek, Vermont, USA) at 450 nm.

Results are expressed as percentage of negativity (PN) compared to the kit control and designated as positive, doubtful or negative according to the cut-off values recommended by the manufacturer (PN ≤ 70 is positive; 70 < PN < 80 is doubtful; PN ≥ 80 is negative). Statistical analyses were performed using a threshold value of 70 that discriminate between positive (PN ≤ 70) and negative (PN > 70) BTV c-ELISA results.

**Statistical analysis. **Statistical analyses were performed using SPSS (Version 16.0; SPSS Inc., Chicago, USA). The association between age (categorical; juvenile, sub-adult and adult), sex (categorical; male vs. female), season (categorical: spring, summer, fall and winter) and abortion with infection were analyzed by Chi-square test and logistic regression. Differences were considered statistically significant when *p* < 0.05.

## Results

Two hundred and three out of 262 sera tested (77.48%, 95% CI: 72.48 - 82.48%) were positive to BTV antibodies. In a total number of 262 samples, there were 208(79.38%) ewes and 54(20.60%) rams, as the Table 1 shows 175 (84.10%) of the ewes and 28 (51.80%) of the rams had antibodies against BTV. Statistically significant differences were evident between sexes (χ^2 ^= 23.79, df = 1, *p *< 0.001). The odds of observation of infection in ewe in comparison with ram was 4.92(95.00% CI: 2.57 - 9.44). There was no statistically significant difference between infection and recent abortion, so that 56 from 65 of recently aborted ewes and 116 from 136 of normally delivered ewes were seropositive to BTV (χ^2^ = 0.02, df = 1, *p* > 0.05). The odds of infection in recently aborted ewes in comparison with normally delivered ewes was 1.07 (95.00% CI: 0.46 - 2.51). 

Differences between age classes were also observed (χ^2 ^= 49.24, df = 2, *p* < 0.001). The odds of infection in adult animals in comparison with juveniles was 12.51(95.00% CI: 5.15 - 30.36), sub-adult animals in comparison with juveniles was 1.86(95.00% CI: 0.77 - 4.48) and adult animals in comparison with sub-adult was 6.72(95.00% CI: 3.27 - 13.79). No statistically significant differences were observed in BTV seroprevalence within seasons (χ^2^=0.34, df = 3, *p *> 0.05), (Table 1).

**Table 1:** Prevalence against BTV antibodies in sheep from southwest in Kohgiluyeh and Boyer-Ahmad province, Iran

**Category**	**Groups**	**Positive**	**Negative**	**Total**
**Sex**	Female	175(84.13%)	33(15.87%)	208(79.39%)
	Male	28(51.85%)	26(48.15%)	54(20.61%)
**Abortion**	Recently aborted	56(86.15%)	9(13.85%)	65(32.34%)
	Delivered normally	116(85.29%)	20(14.71%)	136(67.66%)
**Age**	Juvenile	13(43.33%)	17(56.67%)	30(11.45%)
	Sub-adult	37(58.73%)	26(41.27%)	63(24.05%)
	Adult	153(90.53%)	16(9.47%)	169(64.50%)
**Season**	Spring	45(75.00%)	15(25.00%)	60(22.90%)
	Summer	50(79.37%)	13(20.63%)	63(24.05%)
	Fall	49(77.78%)	14(22.22%)	63(24.05%)
	Winter	59(77.63%)	17(22.37%)	76(29.00%)

## Discussion

Three classifications of BTV status (BTV free zones, BTV seasonally free zones, and BTV infected zones), that affect transportation and free trade of ruminants have been defined.^14^ This study has shown that Kohgiluyeh and Boyer-Ahmad province is considered a BTV infected zone, with BTV infection being highly widespread (77.50%) in this province.

A seroprevalence (34.70%) of BTV infection has been reported in sheep flocks in West Azarbaijan, Iran. In that survey, 172 of 184 flocks were BTV seropositive sheep (93.50%).^15^ The higher seroprevalence obtained in our study compared to the result of Shoorijeh *et al*. could be related to spatial and temperature variations.^14^ Spatial variations observed in seroprevalence among areas may also be due to differences in the distribution of Culicoides vectors.^8^^,^^16^ In the case of temperature, West Azarbaijan province is generally colder than Kohgiluyeh and Boyer-Ahmad areas and this low temperature can affect the existence of colicoides vector that are not able to live at low temperatures. This explanation can be a reason why lower seroprevalence was seen in West Azarbaijan compared to Kohgiluyeh and Boyer-Ahmad.^17^^,^^18^ There is another hypothesis about high seroprevalence of BTV in this area. Pakistan is an eastern neighbor of Iran and high seroprevalance of the BTV infection (48.40%) is reported in Pakistan.^19^ The large volume of animal trade between Iran and Pakistan, especially sheep, could be the reason of high seroprevalance of BTV infection in Iran.

Wild ruminants may play a role in the epidemiology of BTV and they could act as reservoirs in transmission and maintenance of the virus.^20^^,^^21^ The existence of wild ruminants in Kohgiluyeh and Boyer-Ahmad province could have an important influence on the evolution of infection in livestock in this province.

Sheep over one year old (sub-adults and adults) have significantly higher seroprevalences (*p *< 0.001) than juveniles in this study. This is not unexpected, because animals older than 1 year old are likely to have been exposed to the risk of infection for longer than juvenile animals.^22^ Also, higher seroprevalence among adult sheep was likely due to acquired immunity gained over multiple years of exposure to BTV throughout multiple BTV vector seasons.^11^ The seroprevalence in females was higher than males that may be due to the effect of age and sample size.

Nomads in Kohgiluyeh and Boyer-Ahmad province migrate from cold to moderate areas in winter and from warm to moderate areas in summer to maintain stable weather (moderate weather) for their animals throughout the year. This migration and the associated stable weather conditions may be the reason for our results that showed ineffectiveness of season in seroprevalence status. Some of flocks in this study were migrating and those flocks also can be a source of infection of BTV in this province.^23^

Temperature, international trade, geographic status of areas, wildlife characteristic and lifestyle of people are of important factors that all of them can influence prevalence of BTV in the area. Thus, BTV prevalence should be investigated from several perspectives in an area, however, these views are sometimes interrelated and should be taken into consideration altogether and not in isolation. 

In conclusion, this study confirmed that the BTV infection existed in Kohgiluyeh and Boyer-Ahmad province. Since a vaccination program for BT is not established in Iran, a seropositive result indicates BT infection in the domestic populations. According to local weather conditions and facility of vector-borne transmission, prevention and control measures should be considered by health authorities.
